# ALDH1A1-overexpressing cells are differentiated cells but not cancer stem or progenitor cells in human hepatocellular carcinoma

**DOI:** 10.18632/oncotarget.4406

**Published:** 2015-06-22

**Authors:** Kaori Tanaka, Hiroyuki Tomita, Kenji Hisamatsu, Takayuki Nakashima, Yuichiro Hatano, Yoshiyuki Sasaki, Shinji Osada, Takuji Tanaka, Tatsuhiko Miyazaki, Kazuhiro Yoshida, Akira Hara

**Affiliations:** ^1^ Department of Tumor Pathology, Gifu University Graduate School of Medicine, Gifu, Japan; ^2^ Department of Surgical Oncology, Gifu University Graduate School of Medicine, Gifu, Japan; ^3^ Department of Multidisciplinary Therapy for Hepato-Biliary-Pancreatic Cancer, Gifu University Graduate School of Medicine, Gifu, Japan; ^4^ Division of Pathology, Gifu Municipal Hospital, Gifu, Japan; ^5^ Division of Pathology, Gifu University Hospital, Gifu, Japan

**Keywords:** ALDH1A1, hepatocellular carcinoma, prognosis, cancer stem cell marker

## Abstract

Aldehyde dehydrogenase 1A1 (ALDH1A1) is considered to be a cancer stem cell marker in several human malignancies. However, the role of ALDH1A1 in hepatocellular carcinoma (HCC) has not been well elucidated. In this study, we investigated the relationship between ALDH1A1 and clinicopathological findings and examined whether ALDH1A1 deserves to be a cancer stem cell marker in HCC. Sixty HCC samples obtained from surgical resection were collected for immunohistochemical (IHC) staining. Of these 60 samples, 47 samples of HCC tumorous and non-tumorous tissues were evaluated with qRT-PCR. There was no significant difference in the *ALDH1A1*-mRNA level between tumorous and non-tumorous tissues. Tumorous *ALDH1A1*-mRNA level had no relationship with the clinicopathological features. Immunoreactivity of ALDH1A1 was classified into two groups based on the percentage of ALDH1A1-overexpressing cells. The ALDH1A1-high group was significantly associated with low serum levels of α-fetoprotein, small tumor diameter, very little lymphovascular invasion, more differentiated pathology and good stage. The ALDH1A1-high group showed more favorable prognosis for recurrence-free survival. In double-staining IHC, ALDH1A1 was not co-expressed with BMI1, EpCAM, CD13, CD24, CD90 and CD133, which reported as cancer stem cell markers in HCC. In conclusion, ALDH1A1-overexpressing cells could appear to be differentiated cells rather than cancer stem cells in HCC.

## INTRODUCTION

Aldehyde dehydrogenase (ALDH) is a ubiquitous intracellular enzyme that catalyzes the irreversible oxidation of a variety of cellular aldehydes. The ALDH1 family comprises three isoforms (ALDH1A1, ALDH1A2 and ALDH1A3), which synthesize retinoic acid (RA) from the retinal and are crucial regulators for the RA signaling pathway [1]. RA induces gene transcription and thereby modulates a wide variety of biological process like cell proliferation, differentiation, cell cycle arrest and apoptosis [1, 2]. Because of these characteristics of “stemness,” the ALDH1 family is considered to be a stem cell marker [3].

ALDH1A1 particularly serves as a cancer stem cell marker in several human malignancies, although it also serves as a stem cell marker in somatic stem cells such as hematopoietic stem cells and neural stem cells [4, 5]. Several reports discuss the relationship between ALDH1A1 expression level and clinicopathological findings, which may vary depending on the type of cancer present [1, 6–16]. It has been reported that ALDH1A1 expression could be a predictor of poor prognosis in a wide range of cancers such as ovarian carcinoma [6], lung cancer [7], breast cancer [8, 9], esophageal squamous cell carcinoma [10], prostate cancer [11], papillary thyroid carcinoma [12], colorectal carcinoma [13, 14] and gastric cancer [15]. In contrast, it has been reported that ALDH1A1 expression was a favorable prognostic factor in pancreatic cancer [16]. However, the role of ALDH1A1 in hepatocellular carcinoma (HCC) has not been well elucidated. It is still controversial whether ALDH1A1 deserves to be a marker of normal tissue stem cell in liver and cancer stem cells in HCC, and there are several conflicting reports with both affirmative [17, 18] and negative opinions [19] on this issue.

Cancer stem cells (CSCs) are characteristically quiescence, and the dormancy of these small populations protects them from elimination following cancer treatment contributing to cancer relapse [20]. It is important to focus on and eliminate quiescent CSCs in future enhancing long-term cures for cancer patients. In case of HCC, recent immunohistochemical studies of stem cell markers suggest that HCC are histologically heterogeneous and contain a subset of cells expressing a variety of stem cell markers, such as EpCAM [21], BMI1 [22–25], CD13 [26], CD24 [27], CD90 [28] and CD133 [29–32].

In this study, we evaluated the ALDH1A1 expression level in surgical specimens of HCC with two methods, immunohistochemical (IHC) staining and qRT-PCR, and further investigated its relationship with clinicopathological findings and prognosis in HCC patients.

## RESULTS

### Expression level of *ALDH1A1*-mRNA in tumorous and non-tumorous tissues in HCC sections

**Figure 1:**
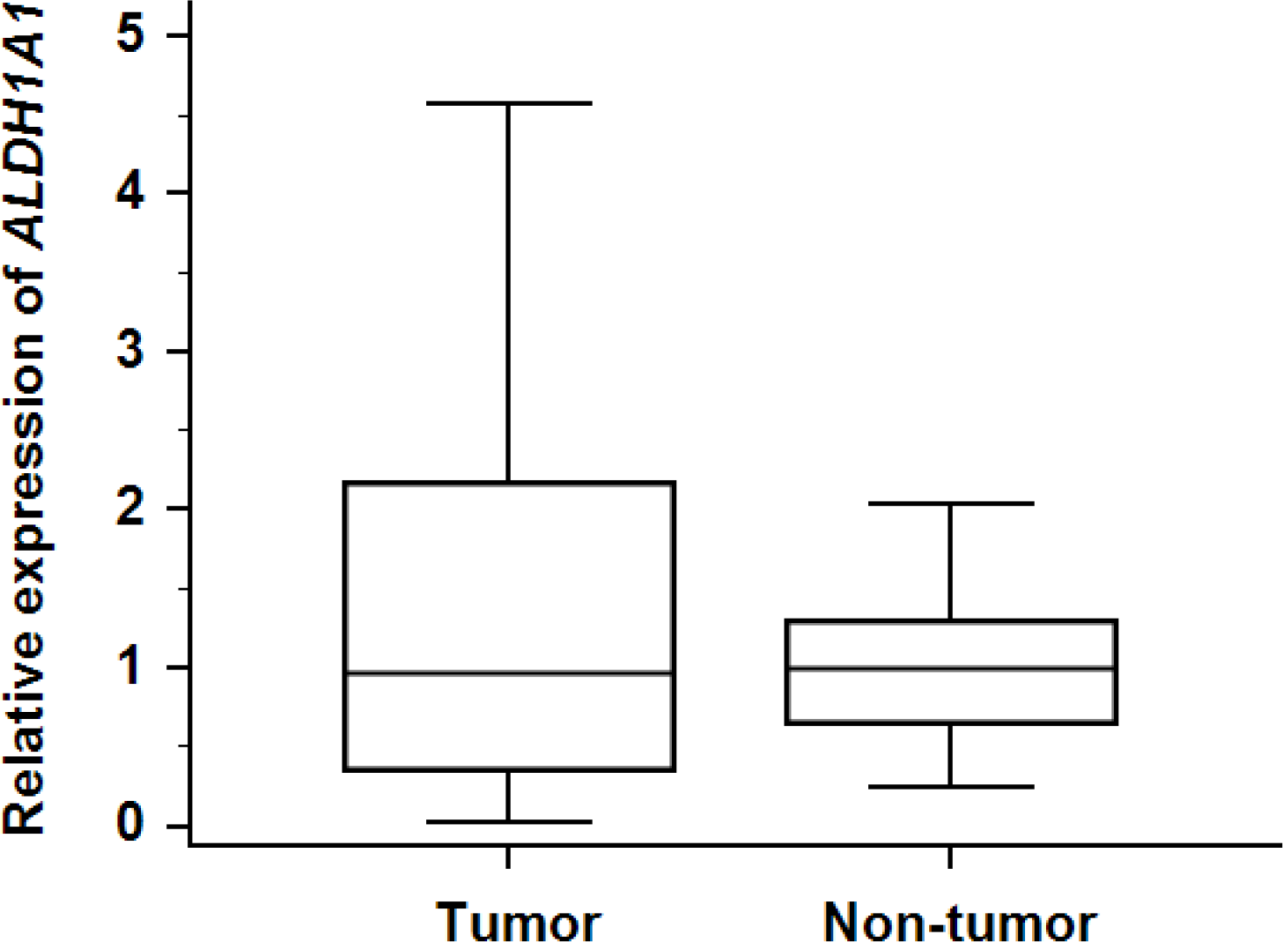
Relative expression of *ALDH1A1*-mRNA in 47 HCC tumorous tissues and corresponding adjacent non-tumorous tissues

**Figure 2:**
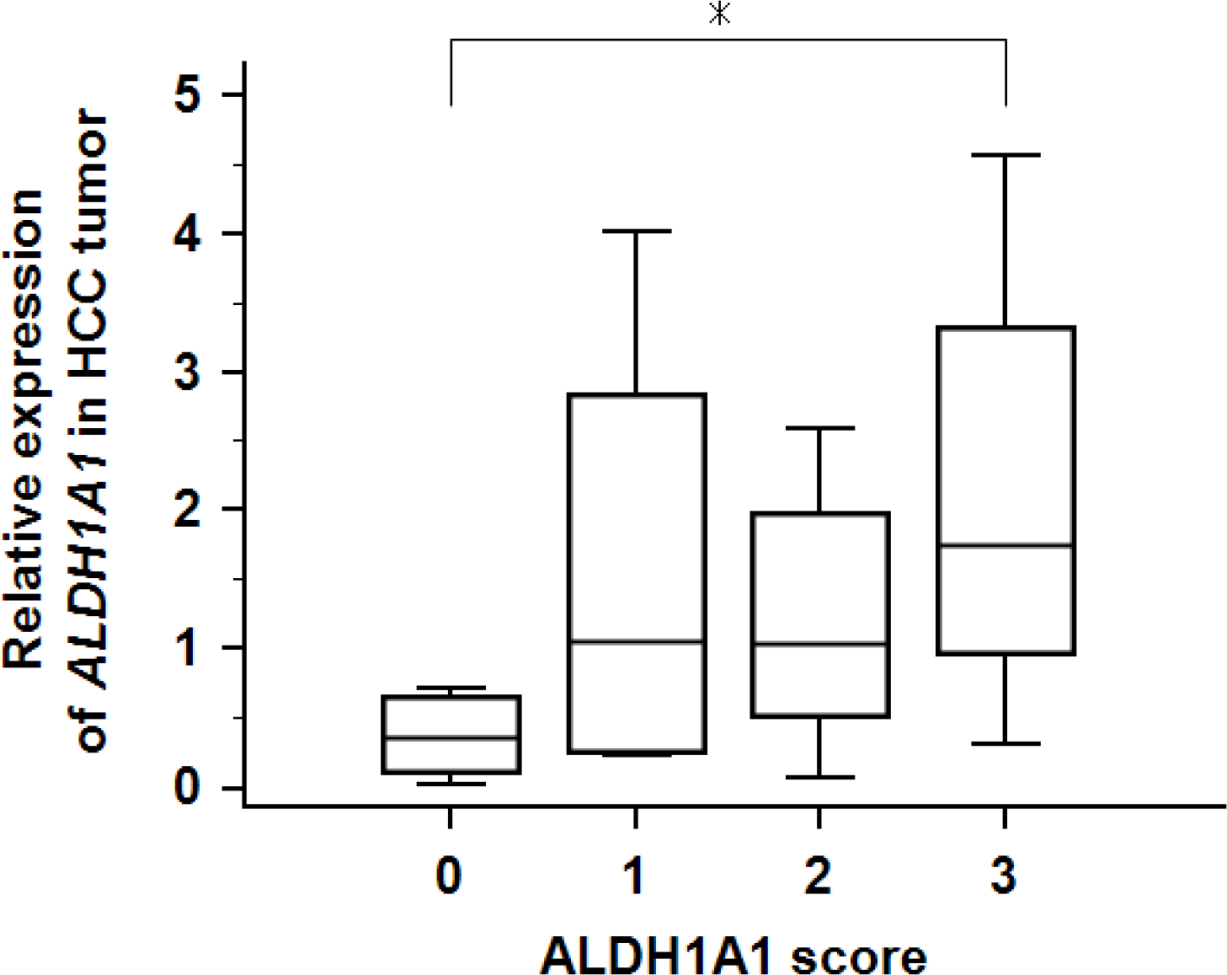
Relative expression of *ALDH1A1*-mRNA in 47 HCC tumorous tissues according to ALDH1A1 score Asterisk (*) indicates *P* < 0.05 as determined by ANOVA.

To investigate whether the transcriptional activity of *ALDH1A1* contributes to HCC carcinogenesis, qRT-PCR assays were performed to determine the transcriptional level of *ALDH1A1* in 47 paired HCC and adjacent non-tumorous tissues. There was no significant difference in the mRNA level of *ALDH1A1* between tumorous and non-tumorous tissues (tumor: 1.36 ± 1.26 vs. non-tumor: 1.00 ± 0.53, *P* = 0.8858) (Fig. 1). According to the ratio of the mRNA expression levels between the tumorous and non-tumorous tissues, the 47 HCC samples were divided into two groups: the *ALDH1A1*-mRNA-low group (in tumor ≤ in adjacent non-tumorous tissue, *n* = 22) and the *ALDH1A1*-mRNA-high group (in tumor > in adjacent non-tumorous tissue, *n* = 25). Of interest, there was no significant difference between the two groups in the clinicopathological features listed in Table 1. For reference, the relative expression of *ALDH1A1* in HCC tissues according to the ALDH1A1 score (described in Materials and Methods) is shown in Fig. 2.

**Table 1:** Clinicopathological features of the ALDH1A1-low group and ALDH1A1-high group (*n* = 60)

	ALDH1A1 expression		*P*
	Low (*n* = 29)	High (*n* = 31)	
Age (years)	66.8 ± 10.6	70.9 ± 8.5	0.1021
Sex (male/female)	21/8	22/9	0.871
BMI	21.7 ± 3.1	22.9 ± 2.8	0.1408
HBV infection (+/−)	7/22	10/21	0.6812
HCV infection (+/−)	13/16	18/13	0.4432
Liver background (Liver cirrhosis/chronic hepatitis/normal)	7/16/2	10/20/1	0.7146
Tumor diameter (mm)	60.9 ± 45.5	29.9 ± 15.1	***0.0019***
V (+/−)	18/11	9/22	***0.0208***
Pathology (wel/wel-mod/mod/mod-por/por)	0/4/19/1/5	7/2/17/4/1	***0.016***
T (1/2/3/4)	4/7/18/0	5/19/7/0	***0.0215***
Stage (I/II/III/IV)	4/7/18/0	5/19/7/0	***0.0215***
UICC stage (I/II/III/IV)	11/18/0/0	22/9/0/0	***0.0208***
HGF (ng/ml)	0.39 ± 0.12	0.39 ± 0.11	0.9085
CEA (ng/ml)	2.9 ± 1.5	4.4 ± 3.6	0.367
CA19-9 (U/ml)	17.3 ± 19.6	34.4 ± 42.4	0.0728
AFP (ng/ml)	6586 ± 17174	998 ± 4101	***0.0168***
PIVKA-II (mAU/ml)	31311 ± 77257	133 ± 3993	0.0719
Albumin (g/dl)	3.7 ± 0.5	3.7 ± 0.4	0.7683
Total bilirubin (mg/dl)	0.77 ± 0.23	0.85 ± 0.30	0.2051

Statistically significant values are displayed in **boldface**.

Abbreviations: BMI, body mass index; HBV, hepatitis B virus; HCV, hepatitis C virus; V, lymphovascular invasion; wel, well differentiated; mod, moderately differentiated; por, poorly differentiated; UICC, Unio Internationalis contra Cancrum; HGF, hepatic growth factor; CEA, carcinoembryonic antigen; CA19-9, carbohydrate antigen 19-9; AFP, α-fetoprotein; PIVKA-II, protein induced by Vitamin K absence or antagonists-II.

### Characteristics and evaluation of ALDH1A1 expression in IHC analysis

**Figure 3:**
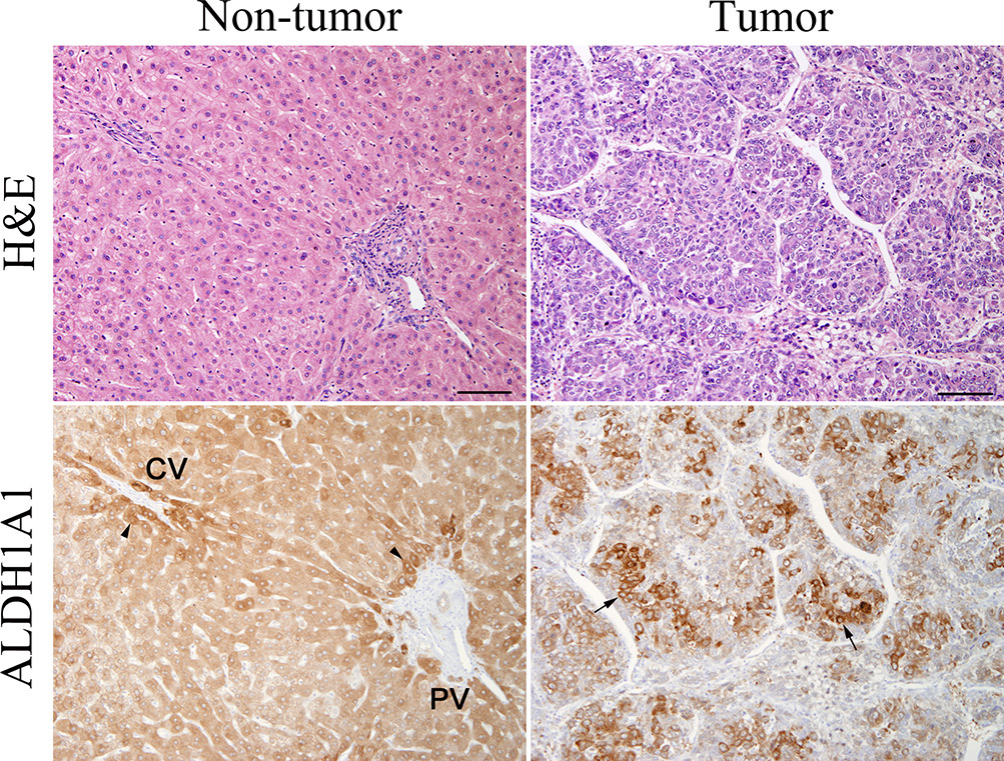
Hematoxylin-eosin (H&E) staining and immunohistochemical staining of ALDH1A1 in tumorous and non-tumorous tissue in HCC ALDH1A1-overexpressing cells (arrows) were defined as more intensely stained cells, compared with perivascular hepatocytes (arrowheads) around central vein (CV) or portal vein (PV) with moderately strong expression. Scale bars indicate 100 μm.

**Figure 4:**
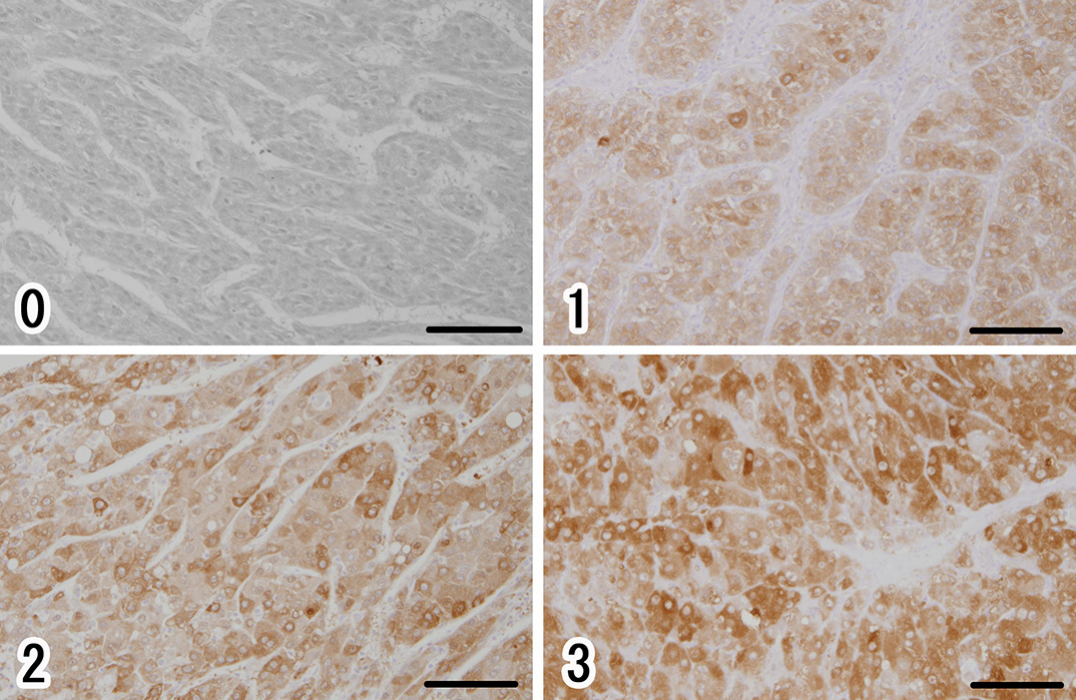
Immunohistochemical evaluation of ALDH1A1 in HCC (0) score 0 (0%), (1) score 1 (1–9%), (2) score 2 (10–20%), (3) score 3 (>20%), according to the percentage of cells strongly expressing ALDH1A1. Scale bars indicate 100 μm.

Next, to investigate the expression level and localization of ALDH1A1 protein, we performed IHC staining with ALDH1A1 antibody. We first confirmed the expression of ALDH1A1 in liver tissues. All non-tumorous tissues in HCC specimens homogenously expressed ALDH1A1 at low levels, with slightly strong expression in the perivascular area around central vein or portal vein, regardless of the various disease backgrounds such as fibrosis or virus infection (Fig. 3). In contrast, HCC tumors contained cells with much higher levels of ALDH1A1 expression, compared to expression levels of perivascular area in non-tumorous tissues, at varying frequencies (Fig. 3). Based on the above characteristics of ALDH1A1 expression, the immunoreactivity of ALDH1A1 was classified as follows based on the percentage of ALDH1A1-overexpressing cells adopted by Suzuki *et al*. [19]: score 0 (0%), score 1 (1–9%), score 2 (10–20%) and score 3 (>20%) according to the percentage of ALDH1A1-overexpressing cells present (Fig. 4). The numbers of HCC samples scored as 0, 1, 2 and 3 were 17, 12, 14 and 17, respectively.

### IHC analysis of ALDH1A1 expression and its relationship with the clinicopathological parameters

We analyzed the relationship between ALDH1A1 expression and clinicopathological characteristics in HCC patients. Pathological evaluation was performed based on the “General Rules for the Clinical and Pathological Study of Primary Liver Cancer” [33] which is in common use in Japan, and tumor differentiation was classified into three stages (wel, mod, por, [+undifferented]) by the degree of cellular and structural atypism, which has been considered to be equivalent of Edmondson classification. Further, based on the scoring system, the primary HCC samples were divided into two groups, the ALDH1A1-low group (score 0 or 1) and the ALDH1A1-high group (score 2 or 3).

The ALDH1A1-high group was significantly associated with low serum levels of α-fetoprotein (AFP) (*P* = 0.0168), small tumor diameter (*P* = 0.0019), very little lymphovascular invasion (*P* = 0.0208), more differentiated pathology (*P* = 0.016) and good stage (*P* = 0.0215) (Table 1).

**Figure 5:**
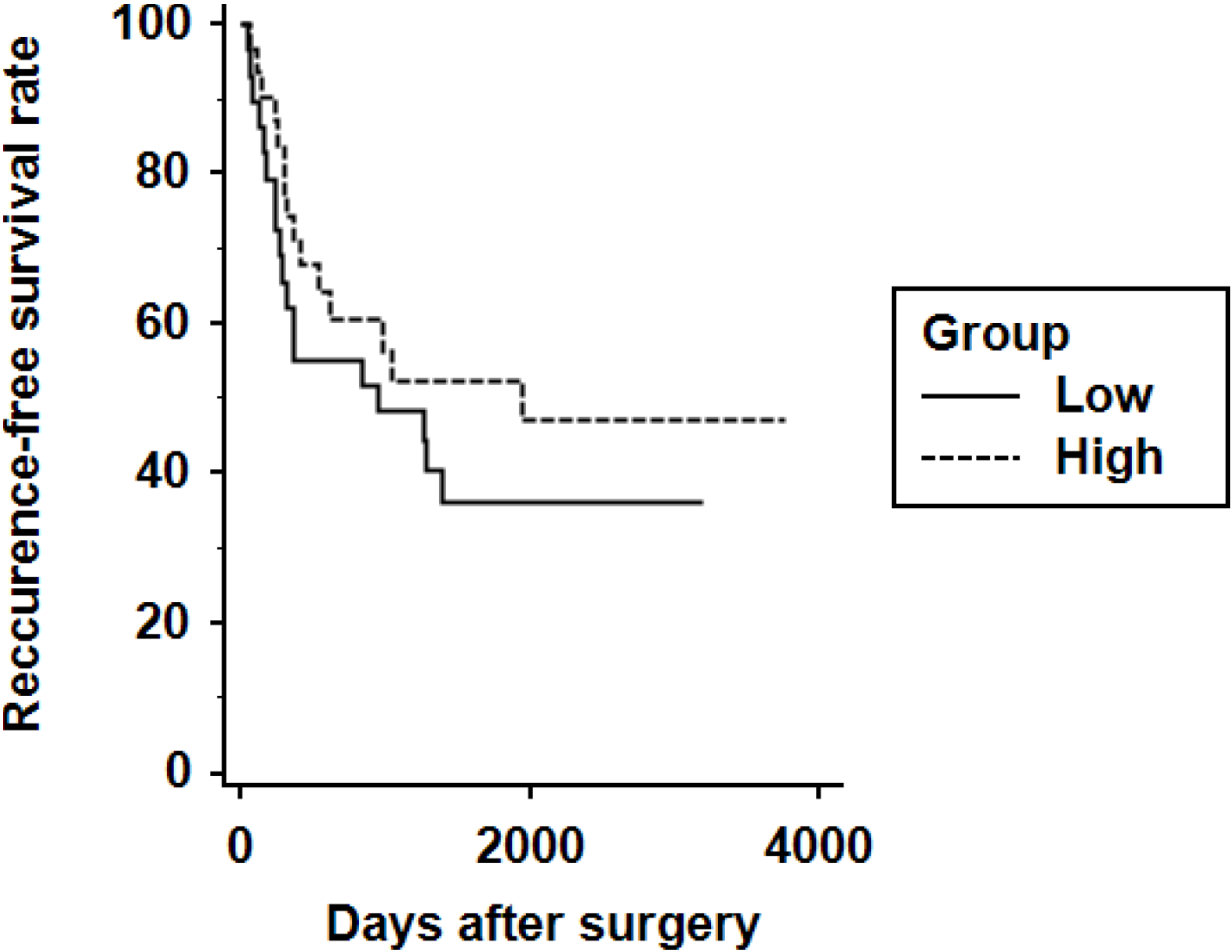
Kaplan-Meier analysis of recurrence-free survival in HCC patients according to ALDH1A1 expression

Next, to perform the prognostic analyses of ALDH1A1 in HCC, survival curves were calculated by Kaplan-Meier method and compared by the log-rank test. During the follow-up period (41.3 ± 37.7 months), 34 patients developed recurrences of HCC, and 9 patients died from HCC. There was no difference between the two groups in overall survival (5-year survival rate was 88.3% in the ALDH1A1-low group vs. 86.4% in the ALDH1A1-high group). Although the ALDH1A1-high group showed a comparatively more favorable prognosis for recurrence-free survival (RFS) than the ALDH1A1-low group, the differences were not statistically significant between the two groups (5-year RFS was 37.9% in the ALDH1A1-low group vs. 54.8% in the ALDH1A1-high group) (Fig. 5).

### Univariate and multivariate analyses of prognostic variables in HCC patients

Univariate and multivariate analyses were performed to identify the effect of ALDH1A1 expression and other clinicopathological parameters on the prognosis of HCC. The variables with *P* < 0.20 on univariate analysis for RFS were subjected to multivariate analysis. Multivariate Cox regression analysis revealed that HCV non-infection (*P* = 0.0375) and low AFP (*P* = 0.0114) correlated significantly with RFS (Table 2).

**Table 2:** Univariate and multivariate analysis of the relative risks for recurrence-free survival in HCC

Variables	Univariate analysis			Multivariate analysis		
	Relative risk	95% CI	*P*	Relative risk	95% CI	*P*
Age (>70 years)	1.432	0.873–2.347	0.1547	1.758	0.744–4.155	0.2006
Sex (male)	1.779	0.898–3.524	0.0986	2.371	0.807–6.966	0.1182
Liver cirrhosis	1.147	0.695–1.895	0.5920			
HBV infection	0.949	0.563–1.597	0.8425			
HCV infection	1.439	0.890–2.328	0.1378	2.457	1.058–5.707	***0.0375***
V (+)	1.299	0.824–2.048	0.2608			
Tumor size (>30 mm)	1.539	0.951–2.489	0.0793	1.503	0.619–3.649	0.3701
Pathology (contain por)	0.446	0.165–1.199	0.1095	0.413	0.108–1.578	0.1981
Stage (≥3)	1.318	0.840–2.067	0.2297			
ALDH1A1 high expression	0.739	0.471–1.158	0.1862	0.871	0.344–2.203	0.7714
AFP (>100 ng/ml)	1.364	0.877–2.119	0.1681	3.502	1.332–9.203	***0.0114***
Albumin (≥3.5 g.dl)	1.143	0.672–1.944	0.6221			
Platelets (≥15 × 104/μl)	1.019	0.644–1.612	0.9376			
Total bilirubin (≥1.0 mg/dl)	1.125	0.686–1.846	0.6412			

Statistically significant values are displayed in **boldface**.

Abbreviations: HCC, hepatocellular carcinoma; CI, confidence interval; HBV, hepatitis B virus; HCV, hepatitis C virus; V, lymphovascular invasion; por, poorly differentiated; AFP, α-fetoprotein.

### ALDH1A1 and cancer stem cell markers are not co-expressed in liver tissue

**Figure 6:**
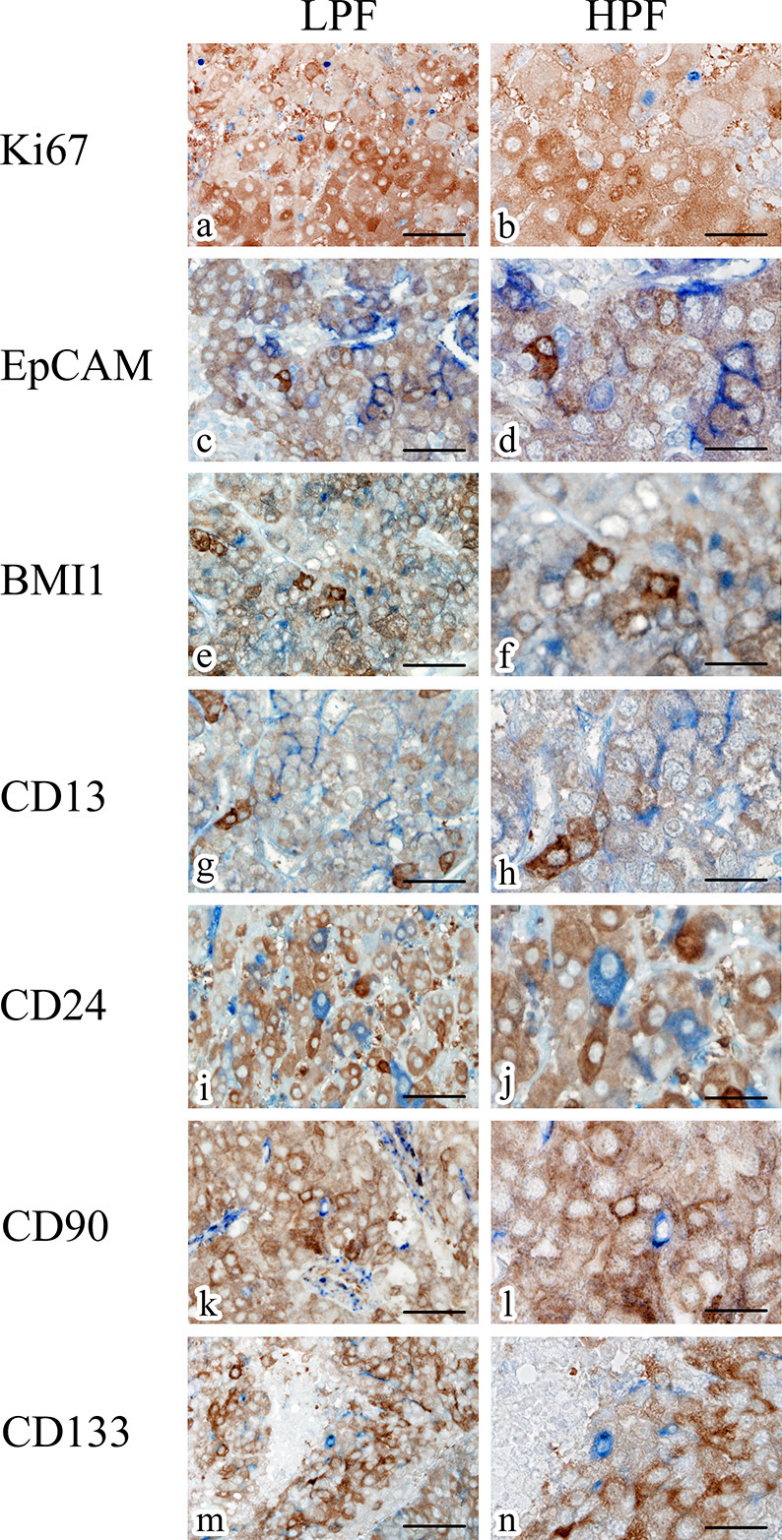
Representative double-staining immunohistochemistry for ALDH1A1 (brown) and Ki67 (blue), EpCAM (blue), BMI1 (blue), CD13 (blue), CD24 (blue), CD90 (blue) and CD133 (blue) in HCC specimens Scale bars indicate 100 μm (a, c, e, g, i, k, m) and 50 μm (b, d, f, h, j, l, n), respectively. Abbreviations: LPF, low-power field; HPF, high-power field.

To examine whether the ALDH1A1-overexpressing cells in HCC have the properties of a cancer stem or progenitor cell, we performed double-staining IHC on randomly selected HCC specimens (Fig. 6). Interestingly, ALDH1A1 was not co-expressed with EpCAM (Fig. 6c), BMI1 (Fig. 6e), CD13 (Fig. 6g), CD24 (Fig. 6i), CD90 (Fig. 6k) and CD133 (Fig. 6m) which have been reported to be cancer stem cell markers in HCC. As well, almost none of the ALDH1A1-overexpressing cells were co-expressed with Ki67 (Fig. 6a), a cell proliferation marker. These data indicate that ALDH1A1-overexpressing cells might not be cancer stem cells in quiescent status or progenitor cells in proliferation status in the HCC stem cell niche. Further, ALDH1A1-overexpressing cells might be differentiated cells, which are in quiescent status for metabolic function of the liver.

## DISCUSSION

Recently, ALDH1A1 could be regarded as a cancer stem cell marker in several epithelial malignancies, and the development of ALDH1A1-targeted therapy is expected. However, it is still controversial whether ALDH1A1 deserves to be a cancer stem cell marker in HCC and there are several conflicting reports. In this study, we evaluated the expression level of ALDH1A1 in primary HCC surgical sections with two methods, IHC staining and qRT-PCR, investigated the relationship between ALDH1A1 and clinicopathological findings, and examined whether ALDH1A1 deserves to be a cancer stem cell marker in HCC. As a result, our study showed a negative finding that ALDH1A1 is not a cancer stem cell marker in HCC, but which could propose as an important data for the development of HCC therapy.

We first examined the mRNA expression levels of *ALDH1A1* in the surgical sections. There was no significant difference in the mRNA level of *ALDH1A1* between the tumorous and non-tumorous tissues. Moreover, there was no significant relationship between the mRNA expression levels of HCC tumorous tissue and clinicopathological features. In contrast, a significant relationship was detected in IHC evaluation between ALDH1A1 and the clinicopathological features. These inconsistent results could be explained by the following reason. In mRNA evaluation, we divided the primary HCC samples into two groups based on the ratio of the mean values of mRNA between the tumorous and non-tumorous tissues. Meanwhile in IHC evaluation, we divided the primary HCC samples into two groups based on the percentage of ALDH1A1-overexpressing cells. Since the evaluation methods are different, there could be the discrepancy in the results between protein by IHC and mRNA levels.

The important thing to note in the present IHC evaluation is that we have focused on ALDH1A1-overexpressing cells. We defined ALDH1A1-overexpressing cells as more intensely stained cells, compared with perivascular hepatocytes, which show moderately strong expression in the surrounding normal liver tissue. We found ALDH1A1 to be expressed very heterogeneously and non-uniformly within the tumor tissue of the HCC specimens. It is unknown whether these ALDH1A1-overexpressing cells are the equivalent of “ALDH-bright cells” [34]. ALDH bright cells can be detected with ALDEFLUOR reagent by using flow cytometry or fluorescent microscopy. ALDH bright cells have been found in cancer tissues including breast, liver, colon and acute myelogenous leukemia. ALDH bright cells are regarded as cancer stem cells based on their proliferation rates, migration and adhesion ability. The metastatic potential of ALDH bright cells is greater than that of ALDH low cells, and ALDH bright cells contribute to cancer chemoresistance. Previous reports on the liver suggested that high ALDH activity evaluated by flow cytometry could be a marker of liver progenitor cells in normal liver [17] and cancer stem cells in HCC [18]. ALDH bright cells are identified by ALDEFLUOR assay based on the enzymatic activity of ALDH1A1. Meanwhile, ALDH1A1-overexpressing cells are identified by IHC based on the localization of ALDH1A1. From the points of difference in cell identification methods or stemness characteristics, we consider ALDH1A1-overexpressing cells are different from ALDH bright cells in HCC.

There is only one report, that by Suzuki *et al*. [19], on IHC evaluation of ALDH1A1 in primary HCC specimens. They adopted the same evaluation method, namely evaluation by the percentage of ALDH1A1-overexpressing cells, and came to the similar conclusion that ALDH1A1-high HCC was significantly associated with low serum levels of AFP, well-differentiated pathology and a favorable clinical outcome. In addition, we further investigated the colocalization between ALDH1A1 and several cancer stem/progenitor markers (EpCAM, BMI1, CD13, CD24, CD90 and CD133) which have discovered recently [21–32] to evaluate “stemness” in ALDH1A1-overexpressing cells in this report. Of interest, but in conflict with previous reports [18], the ALDH1A1-overexpressing cells showed no co-expression with any of these cancer stem cell markers. Considering that the presence of cancer stem cells is generally associated with poor histopathological grade and worse survival [35], our results suggested that ALDH1A1 would not deserve to be called a cancer stem cell marker in HCC.

Several studies reported that ALDH1A1 expression in IHC could correlate with Ki67 expression (indicative of proliferation rate) in pancreas cancer [16] and breast cancer [35]. Conversely, our study revealed that almost none of the ALDH1A1-overexpressing cells were co-expressed with Ki67, which indicates that ALDH1A1-overexpressing cells were not associated with cell proliferation in HCC.

In summary, we focused on the IHC evaluation of ALDH1A1-overexpressing cells. The presence of ALDH1A1-overexpressing cells in high frequency could be a factor indicative of well-differentiated pathology and favorable clinical prognosis. ALDH1A1-overexpressing cells appear to function as a differentiation marker rather than as a cancer stem cell marker in HCC. We should be careful about therapeutic targeting of cancer stem-like or tumor-initiating cells in hepatocellular carcinoma.

## MATERIALS AND METHODS

### Patients and tissue specimens

A total of 60 tumor samples were collected from patients with HCC who underwent surgical resection at Gifu University Hospital between 2004 and 2013. Anatomical hepatic resection or partial hepatectomy was performed in any case and there was no case of liver transplantation. The following patients were excluded: those diagnosed as combined hepatocellular-cholangiocarcinoma, with multiple tumors, with other therapies such as chemotherapy and embolization before surgery, and with extensive necrosis by pathological examination.

The patients comprised 43 men and 17 women with an average age of 68.9 years (range, 40 to 85 years). The follow-up time for these patients ranged from 0 to 122 months with a median follow-up time of 41.3 months. The characteristics of the 60 patients are listed in Table 1.

The 60 HCC samples for IHC staining were fixed in formalin and embedded in paraffin. Of these 60 samples, 47 HCC tumorous tissues and their corresponding adjacent non-tumorous tissues for qRT-PCR were immediately stored at −80°C after surgical resection until use. Informed consent was obtained from each patient.

### Histopathological and immunohistochemical staining

All paraffin-embedded HCC tissues and surrounding non-tumorous tissues were cut into 3-μm-thick serial sections and deparaffinized. These sections were stained with hematoxylin and eosin (H&E) or were used for IHC. For IHC, the sections were placed in citrate buffer (10 mmol^−1^, pH 6.0) and then autoclaved at a chamber temperature of 121°C for 1 min to retrieve the antigen. They were then rinsed and blocked in 3% hydrogen peroxide in methanol for 10 min to remove endogenous peroxidase. Non-specific binding sites were blocked in 0.01 M phosphate-buffered saline (pH 7.4) containing 2% bovine serum albumin (Wako Pure Chemical, Osaka, Japan) for 40 minutes. They were incubated with ALDH1A1 antibody (Rabbit IgG, 1:500; abcam, Cambridge, UK) for 90 min at room temperature. Primary antibodies were detected with a biotinylated anti-rabbit IgG (1:300; Dako, Glostrup, Denmark) for 30 min at room temperature, followed by incubation with avidin-coupled peroxidase (Vectastain ABC kit; Vector Laboratories, Burlingame, CA) for 30 min. The bound complex was visualized using diaminobenzidine (DAB) liquid chromogen (SIGMA, St. Louis, MO) and counterstained with hematoxylin.

For double-staining IHC, Ki67 (1:100; Dako), EpCAM (rabbit IgG, 1:150; abcam), BMI1 (rabbit IgG, 1:200; abcam), CD13 (1:50; Santa Cruz Biotechnology, Santa Cruz, CA), CD24 (1:100; Santa Cruz Biotechnology), CD90 (1:150; Epitomics, Burlingame, CA) and CD133/1 (1:10; Miltenyi Biotech, Bergisch Gladbach, Germany) were used as primary antibodies. Signal amplification was performed with alkaline phosphatase-biotin complex (Vectastain) followed by a chromogenic reaction with alkaline phosphatase substrate kit (Vecta Blue; Vector Laboratories) and DAB. Histological evaluation was performed with the support of two experienced pathologists (H.T. and K.T.) who were blinded to the clinical data.

### Evaluation of immunohistochemical staining of ALDH1A1

HCC tumors contained cells with much higher levels of ALDH1A1 expression, compared to expression levels of non-tumorous tissues. These cells were defined as ALDH1A1-overexpressing cells. Based on the percentage of ALDH1A1-overexpressing cells [19], HCC tissues were classified as follow: score 0 (0%), score 1 (1–9%), score 2 (10–20%) and score 3 (>20%). Moreover, based on the scoring system, HCC tissues were divided in the ALDH1A1-low group (score 0 or 1) and the ALDH1A1-high group (score 2 or 3).

### Real-time quantitative RT-PCR

Frozen tissues were homogenized (Bullet Blender Storm; Chiyoda Science, Tokyo, Japan), and total RNA was extracted using an RNeasy Mini kit (Qiagen, Tokyo, Japan). cDNA was synthesized from 2 μg RNA, using SuperScript III First-Strand Synthesis System for RT-PCR (Invitrogen, Tokyo, Japan) according to the manufacturer's instructions. Real-time quantitative RT-PCR of *ALDH1A1* was performed using SYBR Premix Ex Taq (Takara Bio, Shiga, Japan) on a Thermal Cycler Dice Real Time System (Takara Bio). The expression of *ALDH1A1* was normalized to β-actin expression by using the standard curve method.

Primers for ALDH1A1 and β-actin were designed as follows: ALDH1A1 (forward GCACGCC AGACTTACCTGTC, reverse CCTCCTCAGTTGCA GGATTAAAG) and β-actin (forward GGCCACGGCT GCTTC, reverse GTTGGCGTACAGGTCTTTGC).

### Statistical analysis

Continuous data are presented as the mean ± standard deviation. Statistical differences between two groups were analyzed with the Student *t*-test, the Mann-Whitney *U* test and the chi square test according to the circumstances. Survival curves and RFS were calculated using the Kaplan-Meier method and compared by the log-rank test. The Cox proportional hazard model was used for univariate and multivariate analyses to explore the effects of the clinicopathological parameters and ALDH1A1 expression on RFS. A *P* value of < 0.05 was considered to be statistically significant. Statistical analysis was performed using MedCalc software Ver. 12.4.0 (MedCalc Software, Ostend, Belgium) and StatMate V for Win&Mac Hybrid (ATMS Co., Ltd., Tokyo, Japan).

## References

[1] Marcato P, Dean CA, Giacomantonio CA, Lee PW (2011). Aldehyde dehydrogenase: its role as a cancer stem cell marker comes down to the specific isoform. Cell Cycle.

[2] Koppaka V, Thompson DC, Chen Y, Ellermann M, Nicolaou KC, Juvonen RO, Petersen D, Deitrich RA, Hurley TD, Vasiliou V (2012). Aldehyde dehydrogenase inhibitors: a comprehensive review of the pharmacology, mechanism of action, substrate specificity, and clinical application. Pharmacol Rev.

[3] Marchitti SA, Brocker C, Stagos D, Vasiliou V (2008). Non-P450 aldehyde oxidizing enzymes: the aldehyde dehydrogenase superfamily. Expert Opin Drug Metab Toxicol.

[4] Hess DA, Meyerrose TE, Wirthlin L, Craft TP, Herrbrich PE, Creer MH, Nolta JA (2004). Functional characterization of highly purified human hematopoietic repopulating cells isolated according to aldehyde dehydrogenase activity. Blood.

[5] Pearce DJ, Bonnet D (2007). The combined use of Hoechst efflux ability and aldehyde dehydrogenase activity to identify murine and human hematopoietic stem cells. Exp Hematol.

[6] Landen CN, Goodman B, Katre AA, Steg AD, Nick AM, Stone RL, Miller LD, Mejia PV, Jennings NB, Gershenson DM, Bast RC, Coleman RL, Lopez-Berestein G, Sood AK (2010). Targeting aldehyde dehydrogenase cancer stem cells in ovarian cancer. Mol Cancer Ther.

[7] Alamgeer M, Ganju V, Szczepny A, Russell PA, Prodanovic Z, Kumar B, Wainer Z, Brown T, Schneider-Kolsky M, Conron M, Wright G, Watkins DN (2013). The prognostic significance of aldehyde dehydrogenase 1A1 (ALDH1A1) and CD133 expression in early stage non-small cell lung cancer. Thorax.

[8] Liu Y, Lv DL, Duan JJ, Xu SL, Zhang JF, Yang XJ, Zhang X, Cui YH, Bian XW1, Yu SC (2014). ALDH1A1 expression correlates with clinicopathologic features and poor prognosis of breast cancer patients: a systematic review and meta-analysis. BMC Cancer.

[9] Khoury T, Ademuyiwa FO, Chandrasekhar R, Jabbour M, Deleo A, Ferrone S, Wang Y, Wang X (2012). Aldehyde dehydrogenase 1A1 expression in breast cancer is associated with stage, triple negativity, and outcome to neoadjuvant chemotherapy. Mod Pathol.

[10] Yang L, Ren Y, Yu X, Qian F, Bian BS, Xiao HL, Wang WG, Xu SL, Yang J, Cui W, Liu Q, Wang Z, Guo W (2014). ALDH1A1 defines invasive cancer stem-like cells and predicts poor prognosis in patients with esophageal squamous cell carcinoma. Mod Pathol.

[11] Li T, Su Y, Mei Y, Leng Q, Leng B, Liu Z, Stass SA, Jiang F (2010). ALDH1A1 is a marker for malignant prostate stem cells and predictor of prostate cancer patients' outcome. Lab Invest.

[12] Xing Y, Luo DY, Long MY, Zeng SL, Li HH (2014). High ALDH1A1 expression correlates with poor survival in papillary thyroid carcinoma. World J Surg Oncol.

[13] Xu SL, Zeng DZ, Dong WG, Ding YQ, Rao J, Duan JJ, Liu Q, Yang J, Zhan N, Liu Y, Hu QP, Zhang X, Cui YH (2014). Distinct patterns of ALDH1A1 expression predict metastasis and poor outcome of colorectal carcinoma. Int J Clin Exp Pathol.

[14] Kahlert C, Gaitzsch E, Steinert G, Mogler C, Herpel E, Hoffmeister M, Jansen L, Benner A, Brenner H, Chang-Claude J, Rahbari N, Schmidt T, Klupp F (2012). Expression analysis of aldehyde dehydrogenase 1A1 (ALDH1A1) in colon and rectal cancer in association with prognosis and response to chemotherapy. Ann Surg Oncol.

[15] Li XS, Xu Q, Fu XY, Luo WS (2014). ALDH1A1 overexpression is associated with the progression and prognosis in gastric cancer. BMC Cancer.

[16] Kahlert C, Bergmann F, Beck J (2011). Low expression of aldehyde dehydrogenase 1A1 (ALDH1A1) is a prognostic marker for poor survival in pancreatic cancer. BMC Cancer.

[17] Dolle L, Best J, Empsen C, Mei J, Van Rossen E, Roelandt P, Snykers S, Najimi M, Al Battah F, Theise ND, Streetz K, Sokal E, Leclercq IA (2012). Successful isolation of liver progenitor cells by aldehyde dehydrogenase activity in naive mice. Hepatology.

[18] Ma S, Chan KW, Lee TK, Tang KH, Wo JY, Zheng BJ, Guan XY (2008). Aldehyde dehydrogenase discriminates the CD133 liver cancer stem cell populations. Mol Cancer Res.

[19] Suzuki E, Chiba T, Zen Y, Miyagi S, Tada M, Kanai F, Imazeki F, Miyazaki M, Iwama A, Yokosuka O (2012). Aldehyde dehydrogenase 1 is associated with recurrence-free survival but not stem cell-like properties in hepatocellular carcinoma. Hepatol Res.

[20] Reya T, Morrison SJ, Clarke MF, Weissman IL (2001). Stem cells, cancer, and cancer stem cells. Nature.

[21] Kimura O, Takahashi T, Ishii N, Inoue Y, Ueno Y, Kogure T, Fukushima K, Shiina M, Yamagiwa Y, Kondo Y, Inoue J, Kakazu E, Iwasaki T (2010). Characterization of the epithelial cell adhesion molecule (EpCAM)+ cell population in hepatocellular carcinoma cell lines. Cancer Sci.

[22] Chiba T, Miyagi S, Saraya A, Aoki R, Seki A, Morita Y, Yonemitsu Y, Yokosuka O, Taniguchi H, Nakauchi H, Iwama A (2008). The polycomb gene product BMI1 contributes to the maintenance of tumor-initiating side population cells in hepatocellular carcinoma. Cancer Res.

[23] Zhang R, Xu LB, Yue XJ, Yu XH, Wang J, Liu C (2013). Bmi1 gene silencing inhibits the proliferation and invasiveness of human hepatocellular carcinoma cells and increases their sensitivity to 5-fluorouracil. Oncol Rep.

[24] Ruan ZP, Xu R, Lv Y, Tian T, Wang WJ, Guo H, Nan KJ (2013). Bmi1 knockdown inhibits hepatocarcinogenesis. Int J Oncol.

[25] Fan L, Xu C, Wang C, Tao J, Ho C, Jiang L, Gui B, Huang S, Evert M, Calvisi DF, Chen X (2012). Bmi1 is required for hepatic progenitor cell expansion and liver tumor development. PLoS One.

[26] Haraguchi N, Ishii H, Mimori K, Tanaka F, Ohkuma M, Kim HM, Akita H, Takiuchi D, Hatano H, Nagano H, Barnard GF, Doki Y, Mori M (2010). CD13 is a therapeutic target in human liver cancer stem cells. J Clin Invest.

[27] Lee TK, Castilho A, Cheung VC, Tang KH, Ma S, Ng IO (2011). CD24(+) liver tumor-initiating cells drive self-renewal and tumor initiation through STAT3-mediated NANOG regulation. Cell Stem Cell.

[28] Yang ZF, Ho DW, Ng MN, Lau CK, Yu WC, Ngai P, Chu PW, Lam CT, Poon RT, Fan ST (2008). Significance of CD90+ cancer stem cells in human liver cancer. Cancer Cell.

[29] Ma S, Chan KW, Hu L, Lee TK, Wo JY, Ng IO, Zheng BJ, Guan XY (2007). Identification and characterization of tumorigenic liver cancer stem/progenitor cells. Gastroenterology.

[30] Ma S, Lee TK, Zheng BJ, Chan KW, Guan XY (2008). CD133+ HCC cancer stem cells confer chemoresistance by preferential expression of the Akt/PKB survival pathway. Oncogene.

[31] Fan L, He F, Liu H, Zhu J, Liu Y, Yin Z, Wang L, Guo Y, Wang Z, Yan Q, Huang G (2011). CD133: a potential indicator for differentiation and prognosis of human cholangiocarcinoma. BMC Cancer.

[32] Lingala S, Cui YY, Chen X, Ruebner BH, Qian XF, Zern MA, Wu J (2010). Immunohistochemical staining of cancer stem cell markers in hepatocellular carcinoma. Exp Mol Pathol.

[33] The general rules for the clinical and pathological study of primary liver cancer (1989). Liver Cancer Study Group of Japan. Jpn J Surg.

[34] Allahverdiyev AM, Bagirova M, Oztel ON, Yaman S, Abamor ES, Koc RC, Ates SC, Elcicek S, Baydar SY, Canuto RA (2012). Aldehyde Dehydrogenase: Cancer and Stem Cells. Dehydrogenases.

[35] Ma YC, Yang JY, Yan LN (2013). Relevant markers of cancer stem cells indicate a poor prognosis in hepatocellular carcinoma patients: a meta-analysis. Eur J Gastroenterol Hepatol.

[36] Zhong Y, Lin Y, Shen S, Zhou Y, Mao F, Guan J, Sun Q (2013). Expression of ALDH1 in breast invasive ductal carcinoma: an independent predictor of early tumor relapse. Cancer Cell Int.

